# Disparate outcomes in nonsmall cell lung cancer by immigration status

**DOI:** 10.1002/cam4.3848

**Published:** 2021-03-18

**Authors:** Brittney Chau, Philip HG Ituarte, Ashwin Shinde, Richard Li, Jessica Vazquez, Scott Glaser, Erminia Massarelli, Ravi Salgia, Loretta Erhunmwunsee, Kimlin Ashing, Arya Amini

**Affiliations:** ^1^ Department of Radiation Oncology City of Hope National Medical Center Duarte CA USA; ^2^ Department of Surgery City of Hope National Medical Center Duarte CA USA; ^3^ Department of Medical Oncology City of Hope National Medical Center Duarte CA USA; ^4^ Department of Population Sciences City of Hope National Medical Center Duarte CA USA

**Keywords:** Disparities, Ethnicity, Immigrants, lung cancer, nationality, nonsmall cell lung cancer, NSCLC, race

## Abstract

**Objective:**

The purpose of this study was to evaluate overall survival (OS) outcomes by race, stratified by country of origin in patients diagnosed with NSCLC in California.

**Methods:**

We performed a retrospective analysis of nonsmall cell lung cancer (NSCLC) patients diagnosed between 2000 and 2012. Race/ethnicity was defined as White (W), Black (B), Hispanic (H), and Asian (A) and stratified by country of origin (US vs. non‐US [NUS]) creating the following patient cohorts: W‐US, W‐NUS, B‐US, B‐NUS, H‐US, H‐NUS, A‐US, and A‐NUS. Three multivariate models were created: model 1 adjusted for age, gender, stage, year of diagnosis and histology; model 2 included model 1 plus treatment modalities; and model 3 included model 2 with the addition of socioeconomic status, marital status, and insurance.

**Results:**

A total of 68,232 patients were included. Median OS from highest to lowest were: A‐NUS (15 months), W‐NUS (14 months), A‐US (13 months), B‐NUS (13 months), H‐US (11 months), W‐US (11 months), H‐NUS (10 months), and B‐US (10 months) (*p* < 0.001). In model 1, B‐US had worse OS, whereas A‐US, W‐NUS, B‐NUS, H‐NUS, and A‐NUS had better OS when compared to W‐US. In model 2 after adjusting for receipt of treatment, there was no difference in OS for B‐US when compared to W‐US. After adjusting for all variables (model 3), all race/ethnicity profiles had better OS when compared to W‐US; B‐NUS patients had similar OS to W‐US.

**Conclusion:**

Foreign‐born patients with NSCLC have decreased risk of mortality when compared to native‐born patients in California after accounting for treatments received and socioeconomic differences.

## Abstract 2

Foreign‐born patients with NSCLC have decreased risk of mortality when compared to native born patients in California after accounting for treatments received and socioeconomic differences.

## 1 INTRODUCTION

Multiple studies have demonstrated the impact of race on overall survival (OS) for nonsmall cell lung cancer (NSCLC)^1^, ^2^, ^3^, ^4^, ^5^. These studies have shown that racial minorities such as African Americans have worse OS and 2‐year cancer‐specific survival from early stage NSCLC.^5^ In addition, they found that some racial groups are at higher risk of not receiving curative intent therapy, even though new therapies such as SBRT are now available for treatment of unresectable early‐stage NSCLC.^5^ Studies also found that African Americans and patients with lower socioeconomic status were less likely to receive treatments including surgery, leading to lower survival compared to other population subgroups.^1^, ^2^, ^6^

There is limited data evaluating the effects of both race and nation of origin on OS. One study using the Surveillance, Epidemiology, and End Results (SEER) database suggested that OS may be higher for Hispanic and Asian foreign‐born populations but could not make a definitive conclusion due to problems in death linkage matches caused by inaccurate or missing social security numbers.^7^ In our study, data were collected from the California Cancer Registry (CCR) to evaluate OS outcomes by race, stratified by country of origin in patients diagnosed with NSCLC with the purpose of evaluating whether country of origin impacts survival outcomes. We hypothesized patients born outside of the United States would have worse survival compared to native‐born.

## 2 MATERIAL AND METHODS

### 2.1 Data source and patient selection

**FIGURE 1:**
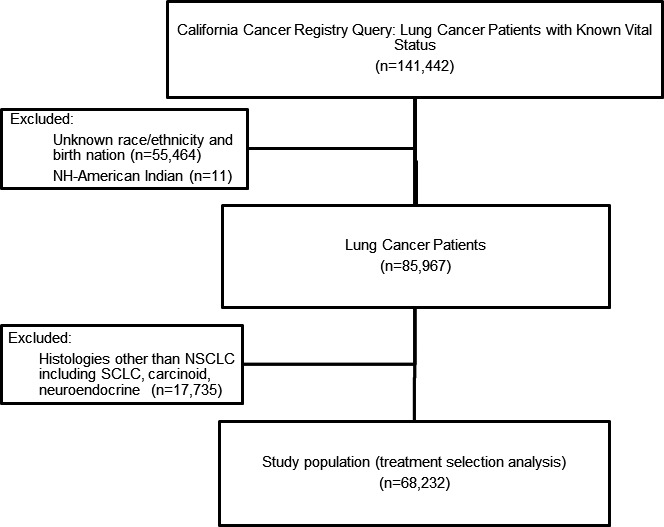
Study schema

Data were obtained on adult cases (age 18 or older) diagnosed with histologically confirmed NSCLC (International Classification of Disease [ICD] for Oncology site codes [third edition] C34.0 to C34.9) recorded in the CCR diagnosed between 2000 and 2012. Patients were included based on the following designated ICD‐O‐3 histologic codes for NSCLC: 8,010, 8,012, 8,013, 8,020, 8,046, 8,050 to 8,052, 8,070 to 8,078, 8,140, 8,141, 8,143, 8,,147, 8,250 to 8,255, 8,260, 8,310, 8,430, 8,480, 8,481, 8,490, 8,560, and 8,570 to 8,575. Patients with small‐cell lung cancer (8,041 to 8,045), carcinoid (8,240), neuroendocrine tumors (8,246), or not otherwise specified (8,000, 8,010) were excluded. NSCLC was the only primary cancer or the first primary cancer for all cases selected. A total of 141,442 patients were queried from the CCR between 2000 and 2012. Patients were excluded if they had other/unknown race/ethnicity and birth nation and those with histologies other than NSCLC (Figure 1). American Indian patients (*n* = 11) were also excluded.

### 2.2 Patient demographics and treatment variables

Potentially relevant patient and treatment characteristics were included. Age was analyzed categorically. Race/ethnicity was classified as eight exclusive groups. The first groups created included non‐Hispanic White (W), non‐Hispanic Black (B), Hispanic (H), and Asian/Pacific Islander (A). This was further stratified by country of origin (US vs. non‐US [NUS]) creating the final eight patient cohorts: W‐US, W‐NUS, B‐US, B‐NUS, H‐US, H‐NUS, A‐US, and A‐NUS. Overall stage was based on the American Joint Committee on Cancer (AJCC) staging guidelines based on corresponding year of diagnosis. The CCR records receipt of definitive surgery, radiation, and chemotherapy which was included in the analysis. Marital status was defined as single or married; those considered separated, divorced, or widowed were categorized as single status. Socioeconomic status, based on the census block group the patient resided in, was divided into five quartiles.^3^, ^4^ Health insurance status was defined as the primary and secondary payer sources and grouped into private insurance, uninsured, or government‐based insurance (Medicare, Medicaid, or any public/military).

### 2.3 Statistical analysis

In accordance with the variables in the CCR registry files, information collected on each patient broadly included demographic, clinical, and treatment data. Sensitivity analyses were performed for patients who were excluded, and no statistically significant differences were seen in the group excluded compared to those included for patient characteristics. Statistical analysis was performed using SPSS V24.0 (SPSS Inc, Chicago, IL). Tests were two‐sided, with a threshold of *p* < 0.05 for statistical significance. First, clinical characteristics of the overall cohort were tabulated. OS time points were calculated from the date of diagnosis to the date of death and were examined using the Kaplan–Meier method. Univariate survival analysis was performed with the log‐rank test. Multivariate Cox regression analysis was performed accounting for patient, disease, and treatment characteristics. Three models were created, one excluding treatment, one including treatment (surgery, radiation, and chemotherapy), and one including treatment in addition to neighborhood socioeconomic status, marital status, and insurance. Proportionality was evaluated for covariates included in the multivariate analysis and returned no significant results.^8^ Multivariable logistic regression analysis was performed to ascertain factors independently associated with patients born in the United States compared to those born elsewhere. Variables included in the multivariable model were selected a priori and based on clinical significance. OR >1 corresponded to higher association with patients born in the United States. The Hosmer–Lemeshow test was used to check for the goodness‐of‐fit of the regression models. To account for immortal time bias, a separate analysis excluding patients who died within 3 months of diagnosis was performed, demonstrating similar results to those presented in this paper (data not shown).

## 3 RESULTS

The total number of patients included was 68,232. The majority of patients were older than 65 (66.3%). The majority of patients were stage IV (46%) at diagnosis, with 18% stage I, 5% stage II, and 24% stage III. When compared to foreign‐born patients, US‐born were more likely older, female, with private insurance, single, presenting with squamous cell carcinoma, earlier stage disease, and appear to more often have received surgery and radiation, and less often chemotherapy (Table 1).

**TABLE 1:** Patient and treatment characteristics

Characteristic	Foreign born		US born		*P*
	No.	%	No.	%	
Age					
<65	6,604	(36.9)	17,440	(34.7)	<0.001
≥65	11,312	(63.1)	32,876	(65.3)	
Gender					
Male	10,138	(56.6)	25,284	(50.3)	<0.001
Female	7,778	(43.4)	25,032	(49.7)	
Race					
NH‐White	3,781	(21.1)	40,550	(80.6)	<0.001
NH‐Black	146	(0.8)	5,403	(10.7)	
Hispanic	4,817	(26.9)	3,460	(6.9)	
Asian	9,172	(51.2)	903	(1.8)	
Insurance status					
Private insurance	5,537	(30.9)	19,074	(37.9)	<0.001
Uninsured	677	(3.8)	942	(1.9)	
Government	10,665	(59.5)	26,927	(53.5)	
Insured‐NOS	522	(2.9)	1,798	(3.6)	
Unknown	515	(2.9)	1,575	(3.1)	
Marital Status					
Single	6,126	(34.2)	23,958	(47.6)	<0.001
Married	11,439	(63.8)	25,551	(50.8)	
Unknown	351	(2.0)	807	(1.6)	
Socioeconomic status					
Lowest	3,140	(17.5)	7,576	(15.1)	<0.001
Lower‐middle	3,368	(18.8)	9,975	(19.8)	
Middle	3,204	(17.9)	10,514	(20.9)	
Upper‐middle	3,253	(18.2)	9,479	(18.8)	
Highest	2,972	(16.6)	8,374	(16.6)	
Unknown	1,979	(11.0)	4,398	(8.7)	
Year of diagnosis					
2000–2003	5,192	(29.0)	18,018	(35.8)	<0.001
2004–2006	4,215	(23.5)	11,868	(23.6)	
2007–2009	4,259	(23.8)	10,787	(21.4)	
2010–2012	4,250	(23.7)	9,643	(19.2)	
Histology					
Adenocarcinoma	9,885	(55.2)	23,183	(46.1)	<0.001
Squamous cell	3,340	(18.6)	11,867	(23.6)	
Large cell/other	4,691	(26.2)	15,266	(30.3)	
Overall AJCC stage					
I	2,824	(15.8)	9,504	(18.9)	<0.001
II	780	(4.4)	2,371	(4.7)	
III	4,097	(22.9)	12,146	(24.1)	
IV	8,963	(50.0)	22,236	(44.2)	
Unknown	1,252	(7.0)	4,059	(8.1)	
Receipt of surgery					
No	13,725	(76.6)	37,047	(73.6)	<0.001
Yes	4,181	(23.3)	13,219	(26.3)	
Unknown	10	(0.1)	50	(0.1)	
Receipt of radiation					
No	11,477	(64.1)	31,338	(62.3)	<0.001
Yes	6,432	(35.9)	18,936	(37.6)	
Unknown	7	(0.0)	42	(0.1)	
Receipt of chemotherapy					
No	9,575	(53.4)	28,996	(57.6)	<0.001
Yes	7,971	(44.5)	20,276	(40.3)	
Unknown	370	(2.1)	1,044	(2.1)	

Abbreviations: AJCC, American Joint Committee on Cancer; CI, confidence interval; HR, hazard ratio; NH, non‐Hispanic; NOS, not otherwise specified; US, United States.

**FIGURE 2:**
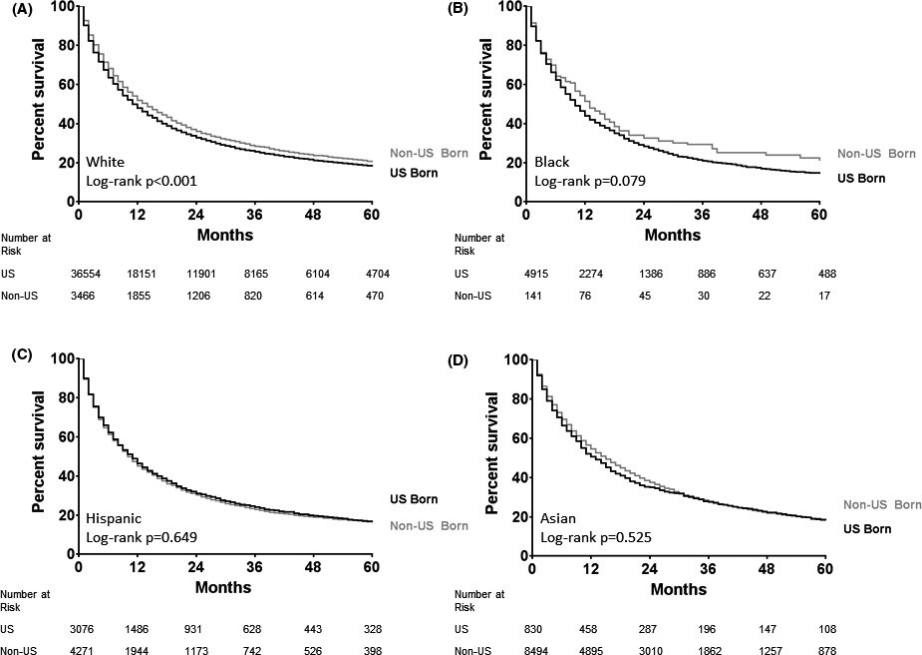
Kaplan–Meier curves of overall survival based on country of origin and stratified by race including White (A), Black (B), Hispanic (C), and Asian (D)

**FIGURE 3:**
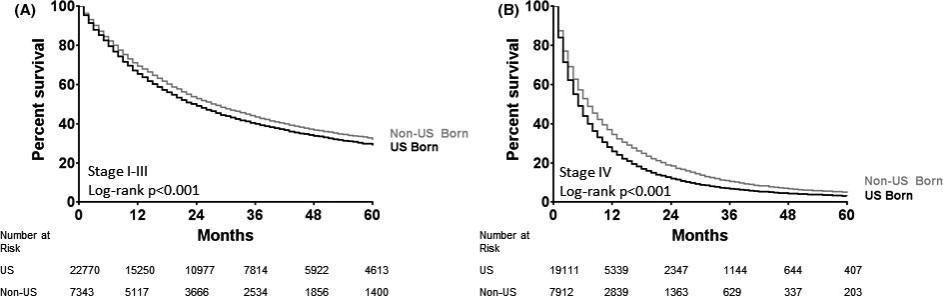
Kaplan–Meier curves of overall survival based on country of origin and stratified by stage I‐III (A) and stage IV (B) disease

Median OS presented from highest to lowest were the following: A‐NUS (15 months), W‐NUS (14 months), A‐US (13 months), B‐NUS (13 months), H‐US (11 months), W‐US (11 months), H‐NUS (10 months), and B‐US (10 months) (*p* < 0.001). Kaplan–Meier curves are shown in Figure 2, comparing the percent survival for US‐born and non‐US‐born patients stratified by race. Unadjusted OS rates appeared similar between native and foreign‐born, stratified by race. Differences were seen favoring W‐NUS over W‐US (*p* < 0.001) with no differences seen in the Black (*p* = 0.079), Hispanic (*p* = 0.649), and Asian (*p* = 0.525) cohorts. When categorized by disease stage, non‐US‐born patients had slightly better OS in both nonmetastatic (median OS 28 vs. 24 months; log rank *p* < 0.001) and metastatic (median OS 7 vs. 5 months; log rank *p* < 0.001) settings (Figure 3).

Compared to W‐US patients, B‐US (HR 1.08, *p* < 0.001), H‐US (HR 1.05; *p* = 0.007), and H‐NUS (HR 1.05, *p* = 0.002) had worse OS. W‐NUS (HR 0.90, *p* < 0.001), A‐US (HR 0.92, *p* = 0.031), and A‐NUS (HR 0.88, *p* < 0.001) had better OS when compared to W‐US. No difference was seen with B‐NUS (HR 0.88, *p* = 0.155). After adjusting for age, gender, stage, year of diagnosis, and tumor histology (Model 1), B‐US had a 6% higher risk of death (HR 1.06, *p* < 0.001) when compared to W‐US, while the other race/ethnicity cohorts had better OS when compared to W‐US, except for H‐US where no statistical significant difference was observed. After further adjustment of treatment received (surgery, radiation, and chemotherapy), B‐US had similar risk of death compared to W‐US (HR 0.98, *p* = 0.141). A‐US, A‐NUS, W‐NUS, and H‐NUS all had better survival outcomes compared to W‐US after accounting for receipt of treatment. In the final adjustment model including neighborhood socioeconomic status, marital status, and insurance, all cohorts had statistically significant better OS when compared to W‐US, except for B‐NUS where there was no difference observed (Table 2).

**TABLE 2:** Univariate and multivariate analysis of predictors of overall survival (OS)

Variable	Univariate						Multivariate					
	HR	95% CI	*P*	HR	95% CI	*P*	HR	95% CI	*P*	HR	95% CI	*P*
				Model 1			Model 2			Model 3		
Race												
W‐US	1			1			1			1		
B‐US	1.08	1.05 to 1.12	<0.001	1.06	1.03 to 1.09	<0.001	0.98	0.95 to 1.01	0.141	0.93	0.90 to 0.96	<0.001
H‐US	1.05	1.01 to 1.09	0.007	1.01	0.97 to 1.05	0.571	0.99	0.95 to 1.02	0.448	0.96	0.93 to 1.00	0.050
A‐US	0.92	0.86 to 0.99	0.031	0.85	0.79 to 0.91	<0.001	0.88	0.82 to 0.95	0.001	0.90	0.84 to 0.97	0.005
W‐NUS	0.90	0.87 to 0.94	<0.001	0.88	0.85 to 0.91	<0.001	0.90	0.87 to 0.93	<0.001	0.91	0.88 to 0.95	<0.001
B‐NUS	0.88	0.73 to 1.05	0.155	0.81	0.68 to 0.98	0.026	0.90	0.75 to 1.08	0.266	0.88	0.73 to 1.06	0.180
H‐NUS	1.05	1.02 to 1.09	0.002	0.96	0.93 to 0.99	0.017	0.89	0.86 to 0.92	<0.001	0.85	0.82 to 0.88	<0.001
A‐NUS	0.88	0.86 to 0.91	<0.001	0.81	0.79 to 0.83	<0.001	0.76	0.74 to 0.78	<0.001	0.77	0.75 to 0.79	<0.001

Note

Model 1: adjusted for age, sex, overall stage, year of diagnosis, and tumor histology. Model 2: model 1 adjusted for surgery, radiation, and chemotherapy. Model 3: model 2 adjusted for neighborhood socioeconomic status, marital status, and insurance.

Abbreviations: A, Asian; B, Black; CI, confidence interval; H, Hispanic; HR, Hazard ratio; NUS, country of origin not United States; US, country of origin United States; W, White.

Unadjusted logistic regression demonstrated that US‐born patients were more likely to be older, female, of higher socioeconomic status, and presenting with squamous cell histology. US‐born patients were more likely to undergo surgery or radiation but less likely to undergo chemotherapy for their disease. Lastly, US‐born patients less commonly were uninsured or had government‐based insurance and less commonly presented with stage IV disease. Under multivariate logistic regression analysis when compared to non‐US‐born, US‐born patients treated for NSCLC in California were again older, more often female, with private insurance, in higher socioeconomic classes, presenting with squamous cell histology, and earlier stage disease. US‐born patients were more likely to receive surgery or radiation and less likely to receive chemotherapy even after accounting for stage (Table S1).

## 4 DISCUSSION

Our results suggest that patients born outside of the US have equal or better survival outcomes compared to patients born in the United States, especially when accounting for receipt of treatment. Race appears to still be a very relevant predictor of OS, supported by prior studies including our study, suggesting that Black patients still struggle with socioeconomic variables that contribute to worse survival outcomes compared to White patients.^9^ For example, African Americans born in the United States continue to have worse OS outcomes, when compared to other races.

Our finding that OS is comparable or better in foreign‐born compared to native‐born patients with NSCLC when accounting for treatment is novel and supported by other smaller series looking at patients treated in California. Patel et al. evaluated Hispanic patients with NSCLC recorded in the CCR and found that foreign‐born Hispanics had a lower rate of disease‐specific mortality compared to those born in the United States, suggesting that this may have been due to ethnic enclaves, which refers to geographic units with dense social networks.^10^ These ethnic enclaves are felt to provide social support enabling better access to care by being less isolated with multiple patient resources enabling them to make multiple visits to hospitals or clinics to receive treatment. Additional studies demonstrate similar results suggesting similar to improved outcomes in survival outcomes in non‐US‐born patients.^11^, ^12^ Based on our findings, in the state of California, survival outcomes in NSCLC are statistically higher for those not born in the United States, though that difference clinically may not be substantial given the overlapping survival curves (Figures 2 and 3).

Another novel finding from our study was that foreign‐born patients were less inclined to undergo surgery and radiation. Additional studies are needed to better understand what treatments are foregone by these patients based on their stage. Overall stage may have contributed as patients born outside the United States were more likely to present with stage IV disease, though this was accounted for in the multivariate model. Other reasons may be cultural factors as some studies have suggested that certain minority groups are less likely to undergo treatment and therefore have higher mortality rates.^6^, ^13^, ^14^ Factors such as fatalistic beliefs and medical mistrust can lead patients away from potentially curative treatments and cause them to be less compliant with follow‐up and receiving treatment.^13^, ^14^, ^15^ Moreover, other types of cancers and diseases demonstrate similar racial disparities. Differences in insurance status, socioeconomic status, and other barriers to healthcare lead to increased mortality in pancreatic, colorectal, and breast cancers.^16^, ^17^, ^18^

Race continues to play a major factor in survival outcomes for NSCLC in California and remains an important disparity that ought to be addressed. Ultimately, steps should be taken to address and resolve the healthcare and racial disparities in treatment, thereby allowing more equal understanding of and access to treatments for patients with NSCLC and other types of cancers and diseases. Studies have demonstrated that physicians using a shared decision‐making model and using supportive communication techniques and hospitals providing cultural competency training can start to bridge the gap in racial disparities in treatment.^19^ Additional solutions such as system‐based intervention and using a real‐time registry with feedback and navigation have shown a decrease in racial disparities and have increased the completion of treatment for minority patients with lung cancer.^20^, ^21^

There are limitations to this study. This is a retrospective study. A large proportion of patients were initially excluded due to unknown race/ethnicity and/or birth nation. B‐NUS also were underrepresented in this study. CCR does not contain critical patient and treatment variables including comorbidities, smoking history, performance scores, and intent of treatment (palliative versus definitive). Further in regards to mixed‐race, the CCR only records one race per patient and is based on what the patient defines themselves to be; mixed‐race is not recorded. In addition, the category defined in this study as NUS consists of multiple countries throughout the world and this therefore represents a fairly heterogeneous population. Further studies are needed to evaluate whether outcomes vary based on continent of birth as an example. Additionally, the CCR also does not record information on recurrences. In this study, socioeconomic status is not patient‐specific but measured at census block group of patient residence. Lastly, molecular markers and use of targeted therapies (oral agents) or checkpoint inhibitors are not recorded in the CCR and are known to be predictive and may vary by ethnicity/race.

## 5 CONCLUSIONS

Using a large, comprehensive state‐wide cancer database of over 65,000 patients diagnosed with NSCLC from 2000–2012, we observed a significant difference in OS in patients born in the United States compared to patients born outside of the United States when accounting for multiple factors including treatment, with foreign‐born patients with NSCLC having decreased risk of mortality when compared to native‐born patients in California. Socioeconomic factors and receipt of treatment play a major role in OS outcomes for those undergoing treatment in California.

## CONFLICT OF INTEREST

The authors report no conflicts of interest.

## AUTHOR CONTRIBUTIONS

Brittney Chau: writing‐original draft, writing‐review, and editing. Philip Ituarte: data curation, writing‐review, and editing. Ashwin Shinde: writing‐review and editing. Richard Li: writing‐review and editing. Jessica Vazquez: writing‐review and editing. Scott Glaser: writing‐review and editing. Erminia Massarelli: writing‐review and editing. Ravi Salgia: writing‐review and editing. Loretta Erhunmwunsee: writing‐review and editing. Kimlin Ashing: writing‐review and editing. Arya Amini: conceptualization, supervision, writing‐original draft, writing‐review, and editing.

ETHICAL APPROVAL.

This article does not contain any studies with human participants or animals performed by any of the authors.

## Supporting information

Table S1

## Data Availability

Research data are not shared.
