# Supplementary material for: Disparate outcomes in nonsmall cell lung cancer by immigration status
Source: Cancer Med. 2021 Mar 18;10(8):2660–7. doi: 10.1002/cam4.3848 (PMC8026917; doi:10.1002/cam4.3848)
Supplement: Supplementary file 1 — Table S1 [file CAM4-10-2660-s001.docx]

| Supplementary Table 1. Logistic Regression Analysis Comparing Lung Cancer Patients Born In the United States to Non-Native Born (Reference) | | | | | | |
| --- | --- | --- | --- | --- | --- | --- |
|  | **Univariate** | | | **Multivariate** | | |
| **Variable** | **OR** | **95% CI** | ***P*** | **OR** | **95% CI** | ***P*** |
| Age |  |  |  |  |  |  |
| <65 | 1 |  |  | 1 |  |  |
| ≥65 | 1.1 | 1.06 to 1.14 | <.001 | 1.16 | 1.11 to 1.21 | <.001 |
| Gender |  |  |  |  |  |  |
| Male | 1 |  |  | 1 |  |  |
| Female | 1.29 | 1.25 to 1.34 | <.001 | 1.17 | 1.13 to 1.21 | <.001 |
| Insurance Status |  |  |  |  |  |  |
| Private Insurance | 1 |  |  | 1 |  |  |
| Uninsured | 0.40 | 0.36 to 0.45 | <.001 | 0.39 | 0.35 to 0.43 | <.001 |
| Government | 0.73 | 0.71 to 0.76 | <.001 | 0.65 | 0.62 to 0.68 | <.001 |
| Insured-NOS | 1.00 | 0.90 to 1.11 | 0.998 | 1.01 | 0.91 to 1.12 | 0.896 |
| Unknown | 0.89 | 0.80 to 0.99 | 0.025 | 0.76 | 0.68 to 0.85 | <.001 |
| Marital Status |  |  |  |  |  |  |
| Single | 1 |  |  | 1 |  |  |
| Married | 0.57 | 0.55 to 0.59 | <.001 | 0.54 | 0.52 to 0.56 | <.001 |
| Unknown | 0.59 | 0.52 to 0.67 | <.001 | 0.59 | 0.52 to 0.67 | <.001 |
| Socioeconomic Status |  |  |  |  |  |  |
| Lowest | 1 |  |  | 1 |  |  |
| Lower-Middle | 1.23 | 1.16 to 1.30 | <.001 | 1.22 | 1.15 to 1.30 | <.001 |
| Middle | 1.36 | 1.28 to 1.44 | <.001 | 1.35 | 1.27 to 1.43 | <.001 |
| Upper-Middle | 1.21 | 1.14 to 1.28 | <.001 | 1.19 | 1.12 to 1.27 | <.001 |
| Highest | 1.17 | 1.10 to 1.24 | <.001 | 1.19 | 1.12 to 1.27 | <.001 |
| Unknown | 0.92 | 0.86 to 0.99 | 0.017 | 1.13 | 1.05 to 1.22 | 0.002 |
| Year of Diagnosis |  |  |  |  |  |  |
| 2000-2003 | 1 |  |  | 1 |  |  |
| 2004-2006 | 0.81 | 0.77 to 0.85 | <.001 | 0.84 | 0.80 to 0.88 | <.001 |
| 2007-2009 | 0.73 | 0.70 to 0.77 | <.001 | 0.76 | 0.72 to 0.79 | <.001 |
| 2010-2012 | 0.65 | 0.62 to 0.69 | <.001 | 0.70 | 0.66 to 0.74 | <.001 |
| Histology |  |  |  |  |  |  |
| Adenocarcinoma | 1 |  |  | 1 |  |  |
| Squamous Cell | 1.52 | 1.45 to 1.59 | <.001 | 1.50 | 1.43 to 1.58 | <.001 |
| Large Cell/Other | 1.39 | 1.33 to 1.45 | <.001 | 1.37 | 1.31 to 1.43 | <.001 |
| Overall AJCC Stage |  |  |  |  |  |  |
| I | 1 |  |  | 1 |  |  |
| II | 0.90 | 0.83 to 0.99 | 0.029 | 0.94 | 0.86 to 1.04 | 0.220 |
| III | 0.88 | 0.83 to 0.93 | <.001 | 0.89 | 0.83 to 0.95 | <.001 |
| IV | 0.74 | 0.70 to 0.77 | <.001 | 0.81 | 0.76 to 0.87 | <.001 |
| Unknown | 0.96 | 0.89 to 1.04 | 0.335 | 0.92 | 0.84 to 1.00 | 0.044 |
| Receipt of Surgery |  |  |  |  |  |  |
| No | 1 |  |  | 1 |  |  |
| Yes | 1.17 | 1.13 to 1.22 | <.001 | 1.11 | 1.06 to 1.18 | <.001 |
| Unknown | 1.85 | 0.94 to 3.65 | 0.075 | 1.27 | 0.58 to 2.78 | 0.557 |
| Receipt of Radiation |  |  |  |  |  |  |
| No | 1 |  |  | 1 |  |  |
| Yes | 1.08 | 1.04 to 1.12 | <.001 | 1.16 | 1.11 to 1.20 | <.001 |
| Unknown | 2.20 | 0.99 to 4.89 | 0.054 | 1.64 | 0.65 to 4.10 | 0.294 |
| Receipt of Chemotherapy |  |  |  |  |  |  |
| No | 1 |  |  | 1 |  |  |
| Yes | 0.84 | 0.81 to 0.87 | <.001 | 0.92 | 0.88 to 0.95 | <.001 |
| Unknown | 0.93 | 0.83 to 1.05 | 0.251 | 0.98 | 0.86 to 1.11 | 0.741 |
| Abbreviations: OR, odds ratio; CI, confidence interval; AJCC, American Joint Committee on Cancer; NOS, not otherwise specified  OR >1 signifies a particular characteristic is more likely to occur in patients born in the United States | | | | | | |
